# Thermocontrolled Reversible Enzyme Complexation-Inactivation-Protection by Poly(*N*-acryloyl glycinamide)

**DOI:** 10.3390/polym13203601

**Published:** 2021-10-19

**Authors:** Pavel I. Semenyuk, Lidia P. Kurochkina, Lauri Mäkinen, Vladimir I. Muronetz, Sami Hietala

**Affiliations:** 1 Belozersky Institute of Physico-Chemical Biology, Lomonosov Moscow State University, 119234 Moscow, Russia; lpk@belozersky.msu.ru (L.P.K.); vimuronets@belozersky.msu.ru (V.I.M.); 2 Department of Chemistry, University of Helsinki, FIN-00014 Helsinki, Finland; Lauri.Makinen@helsinki.fi (L.M.); sami.hietala@helsinki.fi (S.H.)

**Keywords:** thermosensitive polymers, enzyme complexation, controlled release, reversible inactivation, UCST polymers, stimuli-responsive polymers

## Abstract

A prospective technology for reversible enzyme complexation accompanied with its inactivation and protection followed by reactivation after a fast thermocontrolled release has been demonstrated. A thermoresponsive polymer with upper critical solution temperature, poly(*N*-acryloyl glycinamide) (PNAGA), which is soluble in water at elevated temperatures but phase separates at low temperatures, has been shown to bind lysozyme, chosen as a model enzyme, at a low temperature (10 °C and lower) but not at room temperature (around 25 °C). The cooling of the mixture of PNAGA and lysozyme solutions from room temperature resulted in the capturing of the protein and the formation of stable complexes; heating it back up was accompanied by dissolving the complexes and the release of the bound lysozyme. Captured by the polymer, lysozyme was inactive, but a temperature-mediated release from the complexes was accompanied by its reactivation. Complexation also partially protected lysozyme from proteolytic degradation by proteinase K, which is useful for biotechnological applications. The obtained results are relevant for important medicinal tasks associated with drug delivery such as the delivery and controlled release of enzyme-based drugs.

## 1. Introduction

With a growing number of peptide-based and enzyme-based drugs accepted for clinical trials and medicinal use, the development of the approaches for targeted delivery is of special importance. Plenty of approaches such as polymeric nanoparticles or nanogels [1], liposome-based delivery systems [2], protein conjugates, and other nanocarriers [3] have been suggested.

Stimuli-responsive polymers are frequently used as a platform for the construction of new drug delivery systems with an aim at the controlled release of various drugs [4,5,6]. Among such stimuli relevant for biological use, one can mention pH or concentration of specific molecules, light [7,8], and temperature. Thermosensitive polymers provide an opportunity to control the interaction with other macromolecules, especially proteins, by temperature. Thus, the temperature-dependent interaction of polymers with lower critical solution temperature (LCST) with proteins has allowed the construction of artificial chaperones, which are capable of recognizing the unfolded state of the enzymes [9,10]. In addition to actual chaperones, encapsulation or conjugation approaches have been used to immobilize and stabilize various enzymes for catalytic applications [11,12,13,14,15,16]. However, with LCST type of systems, the thermal denaturation of biocomponents at elevated temperature remains an issue. Examples concerning polymers with upper critical solution temperature (UCST) are less numerous and include some techniques with crosslinking stages required for hydrogel or nanoparticle production [17,18,19]. A simple noncrosslinking cooling-induced protein capturing by UCST-type polymers was suggested as an approach for protein extraction with some specificity to protein charge [20]. Noteworthy, many of such polymers are nontoxic and are prospective for biological use [21,22].

In the present study, we tested the interaction of UCST-type polymer, poly(*N*-acryloyl glycinamide) (PNAGA) [23,24,25], which was already suggested for medicinal use [21,26], with lysozyme as a model enzyme at different temperatures. Lysozyme is an enzyme of the hydrolase class that cleaves to the peptidoglycan component of bacterial cell walls, which leads to cell death; therefore, it is widely used as an antimicrobial agent. We have demonstrated the reversible binding of PNAGA and lysozyme at low temperatures followed by a dissociation after heating that can be used as a platform for the creation of a protein delivery system with controlled release. A key advantage of this strategy is that complexation is accompanied by reversible enzyme inactivation and protection from proteolytic digestion; the heating-induced release of the enzyme is accompanied by its fast reactivation.

## 2. Methods

### 2.1. Materials

**Scheme 1:**
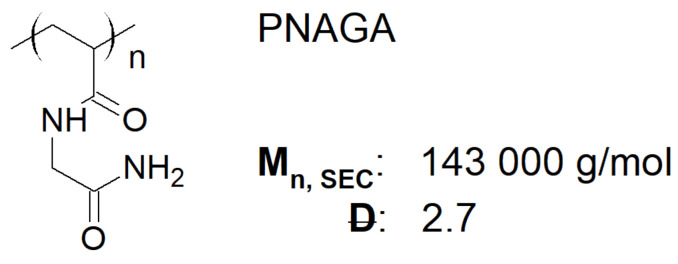
Structure and characteristics of the polymer.

For NAGA, monomer synthesis glycinamide hydrochloride (Bachem, Bubendorf, Switzerland) and acryloyl chloride (Sigma-Aldrich, Saint Louis, MO, USA) were used as received. The initiator 2,2′-azobis[2-(2-imidazolin-2-yl)propane] dihydrochloride (VA-044, Wako Specialty Chemicals) was recrystallized from methanol. The monomer and polymer syntheses have been reported earlier by [25]. In brief, polymerizations of NAGA were carried out using a thermal radical initiator VA-044 in DMSO at 60 °C. The structure and main characteristics of the polymer are shown in Scheme 1.

Chicken egg lysozyme was purchased from Sigma-Aldrich. Protein concentration was measured spectrophotometrically using A_280_^0.1%^ value of 2.6 (for sample preparation) and by measuring SDS-PAGE bands intensity (for analysis of the complex composition).

All experiments were performed in 10 mM potassium phosphate-buffered saline, pH 7.4.

### 2.2. Dynamic Light Scattering

The phase-transition behavior of the polymer and its complexes with lysozyme was studied using dynamic light scattering with the ZetaSizer NanoZS instrument (Malvern, UK). The PNAGA solution with a concentration of 10 mg/mL in the absence as well as in the presence of lysozyme with a concentration of 5 mg/mL was incubated overnight on ice before the measurements. The samples were heated up in the instrument with an average heating rate of 0.7°/min to 45 °C and then cooled down with the same rate. Each point was determined as an average over three runs. Temperature of the cloud point was estimated as a temperature of inflection point from a sigmoidal fitting of the curves.

### 2.3. Isothermal Titration Calorimetry

ITC experiments were performed using a VP-ITC calorimeter (MicroCal, Northampton, MA, USA) at 10 and 25 °C. A solution of the polymer (0.8 or 2 mg/mL) was titrated by successive 20 μL injections of lysozyme solution (3 or 2 mg/mL), with a time interval between the injections of 5 min. To compare the heat effect with a heat effect of the dilution of the polymers, the same polymer solutions were titrated with the buffer without lysozyme. All samples were degassed before the experiment. The binding isotherms were fitted with the “one set of sites” model using MicroCal Origin 7.0 software. For the fitting, the concentration of PNAGA was expressed in terms of the molar concentration of NAGA groups.

### 2.4. Preparation of the Complexes

**Figure 1:**
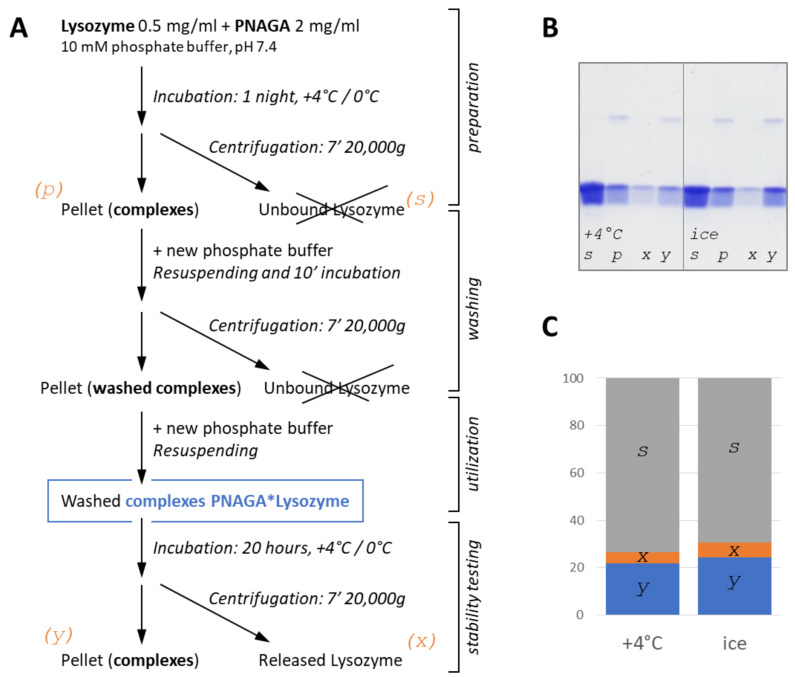
(**A**) The scheme of a simple procedure of mixing and cooling down followed by washing used to prepare stable PNAGA*Lysozyme complexes, as well as an additional step to test the complex stability. (**B**) SDS-PAGE of supernatant (s) and pellet (p) of the first centrifugation step, and supernatant (x) and pellet (y) of the last centrifugation after a stability test for complexes of PNAGA with Lysozyme obtained at +4 and at 0 °C (on ice). (**C**) Amount of lysozyme in the above samples determined using Bradford protein assay and expressed as a percentage of total amount of lysozyme.

The stable PNAGA*Lysozyme complexes were prepared using the following simple procedure (Figure 1A). The enzyme and the polymer solutions were mixed at room temperature in 10 mM phosphate buffer, pH 7.4, and cooled down to +4 or 0 °C (i.e., on ice). After overnight incubation, the formed complexes were separated from unbound lysozyme by centrifugation and washed with pure phosphate buffer. Amount of the bound and unbound protein was measured using Bradford protein assay. For testing complex stability, the washed complexes were incubated for 20 h in pure 10 mM phosphate buffer, pH 7.4 and separated from released protein in the same manner. All experiments were performed at least three times to obtain statistical data.

### 2.5. Lysozyme Activity Assay

Enzymatic activity of lysozyme was determined from a decrease in absorbance of cell suspension due to addition of the enzyme. The *E. coli* SupF cells treated by freeze were used as a substrate. Sample aliquots containing 0.2–1 μg of lysozyme were mixed with 150 μL of cell suspension, and optical density was measured at 400 nm for 2 min using a VersaMax microplate reader (Molecular Devices, San Jose, CA, USA). Negative control (buffer without enzyme) was subtracted from sample measurements. The activity values were determined as a slope of linear part of the time dependence and then divided by actual lysozyme concentration determined from SDS-PAGE bands intensity. The values were averaged among at least three measurements and expressed as a percentage from the specific activity of free lysozyme at 25 °C.

### 2.6. Proteinase K Proteolysis Assay

Proteolysis was initiated by the addition of 2.5 μL proteinase K (Eurogene, Moscow, Russia) to a concentration of 67, 42, 26, 16, and 10 μg/mL into 20 μL aliquots of sample (PNAGA*Lysozyme complexes). Lysozyme solution with a concentration of 0.1 mg/mL was used as a control. Proteolysis was performed at 4 °C and quenched after 4 h incubation by addition of 1 mM phenylmethylsulfonyl fluoride in isopropanol. The samples were separated on 16% SDS-PAGE. The amount of intact lysozyme was determined from the SDS-PAGE bands intensity using ImageJ software and expressed as a percentage from an initial value.

As an additional control for a possible effect of the polymer on proteinase K activity, the same experiment was performed in 50 mM Tris-HCl buffer, pH 7.4.

## 3. Results

### 3.1. Polymer-Enzyme Complexes Formed by the Mixture Cooling Are Stable in Cold but Dissolute When Heated

**Figure 2:**
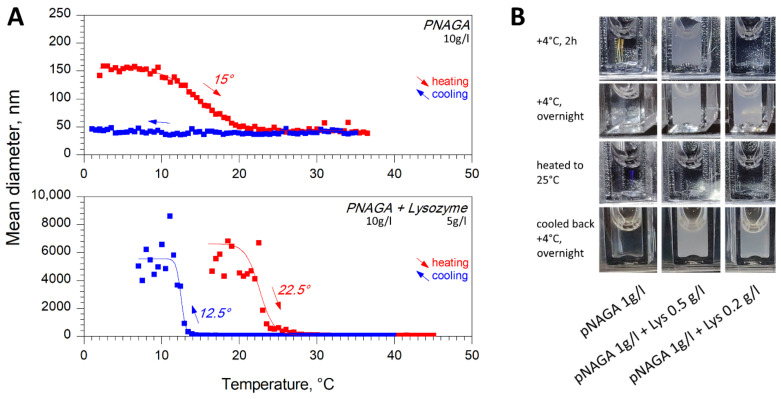
Phase-separation behavior of PNAGA in a solution is altered in the presence of lysozyme. (**A**) Mean diameter of particles determined using DLS for heating (red curves) and cooling (blue curves) of free polymers solutions (top) and their mixtures with lysozyme (bottom). Temperature values of cloud point are presented near the curves. Here, 10 mM phosphate buffer, pH 7.4. (**B**) Cooling down of PNAGA and lysozyme mixtures results in reversible formation of large complexes and decrease in the system transmittance; cooled solutions of free polymers are almost transparent.

The thermosensitive polymer with upper critical solution temperature, namely, poly(*N*-acryloyl glycinamide) homopolymer (PNAGA), was tested for interaction with lysozyme, selected as a model enzyme. The synthesis of the PNAGA polymer used in this study has been reported earlier [25], and its relevant characteristics are reported in Scheme 1. The phase-transition behavior of the 10 mg/mL polymer solution is shown in Figure 2A: a soluble form with the particles diameter of 43 nm at room temperature but larger particles (~160 nm) in cold were detected. The temperature of phase transition for the heating of the precooled sample was 15 °C. As for a cooling experiment, an increase in the particle diameter was not observed, indicating that aggregation is slow. The hysteresis of phase transition is generally observed for PNAGA and is considered to be related to the kinetics of formation of the hydrogen bond network [23,24]. In the presence of 5 mg/mL lysozyme, the aggregation behavior and phase-transition temperature of the polymer were significantly altered: the phase-transition temperature increased up to 22.5 and 12.5° for heating and cooling, respectively; the size of the particles in the cold became much higher (Figure 2A). Since a solution of free lysozyme does not show any phase transition in this temperature interval, the observed effect of lysozyme on the behavior of the polymer clearly indicates interaction between the polymer and the enzyme. However, after a heating up, the preincubated in the cold mixture of PNAGA and lysozyme demonstrated disaggregation and became transparent again.

The data of light scattering agreed well with the visual observation of the systems with lower concentrations of lysozyme and PNAGA, which are more suitable for handling enzymes (Figure 2B). The mixtures of the polymer and lysozyme as well as a solution of free polymer were transparent at 25 °C. However, after a 2 h incubation at +4 °C, the mixture of PNAGA (1 mg/mL) with lysozyme (0.5 mg/mL) became turbid, in contrast to the solution of free polymer and the mixture of PNAGA (1 mg/mL) with lysozyme (0.2 mg/mL). Overnight incubation caused both mixtures of the polymer and lysozyme to become turbid (the system with a higher concentration of the lysozyme became more turbid), whereas free polymer solution was just slightly turbid. Since the solution of free lysozyme is completely transparent, the difference between free polymer and its mixtures with lysozyme indicates the binding of the polymer and lysozyme and formation of complexes, which are larger than particles formed by free PNAGA, which collapsed due to phase transition. When heated back up, all systems became transparent again, suggesting the dissolution of the complexes. Such a cooling–heating cycle can be repeated with the same result.

### 3.2. PNAGA Binds Lysozyme Only at LOW Temperature

**Figure 3:**
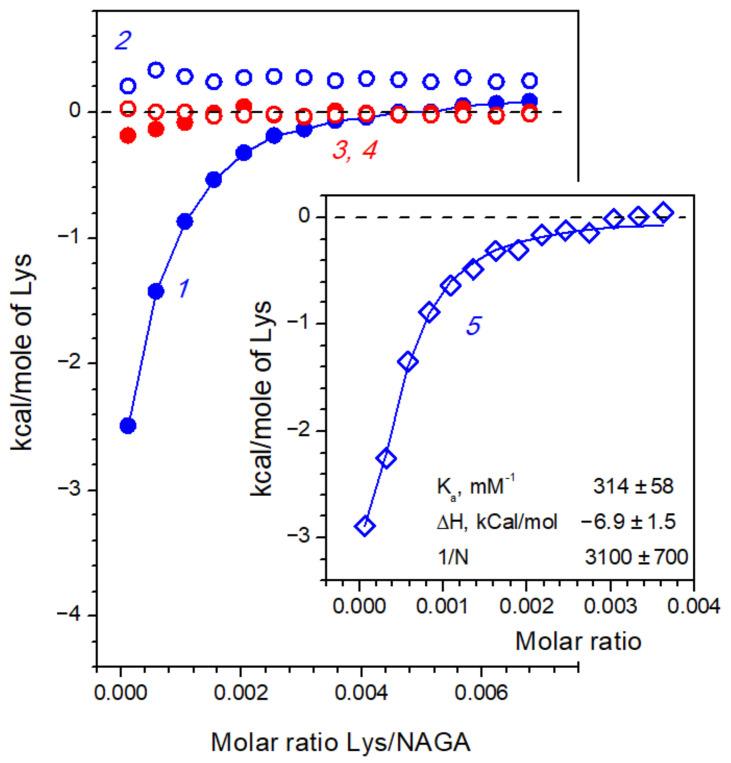
PNAGA binds lysozyme at 10 °C (blue circles) but does not bind it at 25 °C (red circles). ITC data for titration of polymer solutions with lysozyme solutions (curves 1 and 3, filled circles) and buffer solutions (curves 2 and 4, empty circles). The inset represents titration with lower molar ratio and the values of binding constant (K_a_), enthalpy (∆H), and stoichiometry (1/N, in terms of bound NAGA units per a protein molecule) of the binding. Polymer concentration is expressed in terms of molar concentration of NAGA repeated units. 10 mM phosphate buffer, pH 7.4.

The binding of lysozyme with PNAGA polymer was tested directly at different temperature using isothermal titration calorimetry. The polymer efficiently binds lysozyme at 10 °C but does not bind it at 25 °C (Figure 3; compare curves with filled and empty circles, which represent the titration of the polymer solution with a protein solution and buffer solution, respectively). The binding is exothermic process (binding enthalpy < 0) with the binding constant of 3.1 ± 0.6 × 10^5^ M^−1^; the stoichiometry is 3100 ± 700 NAGA monomers per one protein molecule. Such a high value indicates that few polymer chains (in average, 2–3 chains) bind to one protein molecule.

### 3.3. Lysozyme in the Complexes Is Inactive

Based on the presented results, a simple procedure was used to prepare and separate stable PNAGA*Lysozyme complexes (Figure 1B). In brief, solutions of the enzyme and the polymer were mixed at room temperature, cooled down to +4 or 0 °C (i.e., on ice), and incubated overnight. Then, the formed complexes were separated from unbound lysozyme by centrifugation and washed with pure phosphate buffer. Although most of the protein remained unbound, some amount of the lysozyme was captured by the polymer (Figure 1B,C). The complexes obtained at 0 °C (on ice) contain a larger amount of the protein compared to those obtained at +4 °C. The prepared complexes are stable and therefore are appropriate for further usage. Although a 20 h incubation in pure phosphate buffer resulted in the release of a small amount of lysozyme, most of it remained bound (Figure 1B,C).

**Figure 4:**
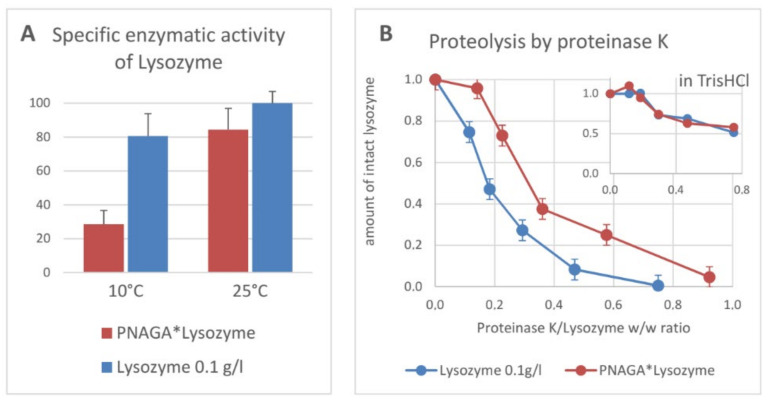
(**A**) Specific enzymatic activity of lysozyme in a free form and complexed with PNAGA. (**B**) Proteolytic digestion of lysozyme by proteinase K. Amount of intact lysozyme determined from SDS-PAGE bands intensity versus protease/lysozyme *w*/*w* ratio; red and blue line for complexes and free lysozyme, respectively. Here, 10 mM phosphate buffer, pH 7.4, +4 °C. Inset represents control experiments in 50 mM TrisHCl buffer, pH 7.4.

The effect of complexation on enzymatic activity of lysozyme (i.e., lysis of bacterial cells) was analyzed (Figure 4A). In the cold, where the prepared complexes PNAGA*Lysozyme are stable, the specific enzymatic activity was about 35% of specific activity of free lysozyme, whilst heating to 25 °C followed by release of the enzyme from the complexes resulted in its almost complete reactivation.

### 3.4. Encapsulation Protects Lysozyme from Proteolytic Degradation

Encapsulated into the complexes with PNAGA, lysozyme was shown to be partially protected from proteolytic cleavage by proteinase K (Figure 4B). The prepared complexes PNAGA*Lysozyme incubated for 4 h at +4 °C in the presence of different concentrations of proteinase K were digested by a significantly lower extent compared to free lysozyme at a similar concentration. To check if the polymer can affect the activity of proteinase K, a similar control experiment was performed in the Tris-HCl buffer, where large complexes of PNAGA and lysozyme are not formed. No effect of the polymer on the proteolysis level was observed (Figure 4B, inset). Thus, the data clearly indicate that the decrease in a proteolysis level is a direct protection of the lysozyme inside the complexes but not an inhibition of the protease by the polymer.

## 4. Discussion

**Figure 5:**
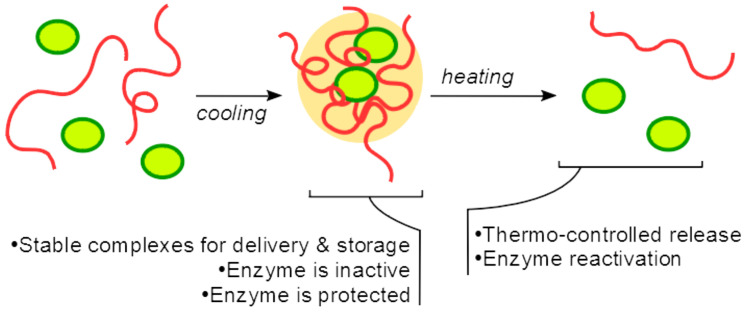
The scheme represents two key advantages of the suggested approach: simple preparation of the complexes with inactivated and protected enzyme and temperature-controlled release of the enzyme accompanied by its reactivation.

To summarize, a prospective technology for reversible enzyme complexation accompanied with its inactivation and protection followed by the reactivation after a thermocontrolled release was demonstrated (Figure 5). A thermosensitive polymer with upper critical solution temperature, poly(*N*-acryloyl glycinamide), was shown to bind lysozyme at cold and to do not bind it at room temperature. Since the binding is reversible, the cooling–heating cycle allows for forming the complexes and performing a controlled release of the enzyme. Noteworthy, the complexes are easy to prepare by a simple mixing of two solutions at room temperature followed by cooling down to the binding temperature and washing of the complexes at cold. No crosslinking steps that might (directly or indirectly due to further purification procedures) affect enzyme structure and activity are required. Thus prepared, the complexes are stable in cold and can be easily handled.

Inside the complexes, lysozyme is inactive, but its enzymatic activity is restored after release from the complexes. It provides an opportunity for reversible and controlled switching off/on the activity of the enzyme. The reversible switching off of the enzyme activity in complex with synthetic polymers was earlier shown in a few papers [27,28], but the release of the enzyme from the complexes was managed by additional polymers, which might complicate the use of such an approach in multicomponent systems. In such systems, the heating-induced enzyme release seems to be preferable since it is gentler. One more advantage of the suggested strategy is a combination of controlled reversible protein inactivation and its partial protection from proteolytic degradation that should facilitate storage of the protein. It can be relevant for the biotechnological use of enzymes at elevated temperature. Thus, a specific enzyme can be stored at a low temperature for a long time in a complexed form (inactive and stabilized) but released from the complexes and performed catalysis when transferred to the system at an elevated temperature (addition of the complexes into bioreactor system or direct heating of the system, which initially contains the complexed enzyme).

Temperature-controlled release is relevant for some important medicinal tasks associated with drug delivery. Indeed, the enzyme inside the complexes is inactive and partially protected from proteolytic degradation but can be easily reactivated due to controlled release. Focused on biological approaches, we used physiological buffer system, namely potassium phosphate buffer, pH 7.4. Of course, our model system with phase-transition temperature lower than 25 °C looks to be not optimal for medicinal usage, although some tasks, including skin delivery, allow for system formulation in a very wide temperature range due to a high ability to cooling without the occurrence of undesired side effects [29,30]. Regardless, the temperature of phase transition of PNAGA-based polymers can be tuned in a wide range by copolymerization or by adjusting the molar mass or end-groups [31,32,33,34,35]. For example, using a hydrophobic dodecyl end-group in RAFT polymerization of NAGA and changing the molar mass, the phase transition upon heating could be tuned in the range from 24 to 43 °C [32], which covers physiologic temperature range. Copolymeric PNAGA-poly(*N*-phenylacrylamide) brushes, which sorb and release cells at 30 and 37 °C, respectively, can be one more example [35]. A fine-tuning of the temperature of the transition allows one to perform a selective release of a complexed enzyme only in particular organs or tissues, the temperature of which increased due to pathologies such as inflammation, and to preserve the complexes stable (and inactive) in healthy tissues. Unfortunately, it might be difficult to obtain such a fine selectivity since the difference between the temperatures of normal and inflamed tissues is small, namely fewer than a few degrees. However, the selective heating of specific regions and tissues (in particular, cancer tumors) can be achieved using lasers as well as a radio frequency radiation combined with gold or silicon nanoparticle-based sensitizers [36]. We suppose that a combination of selectively induced hyperthermia and the introducing of thermoresponsive polymer–enzyme complexes, which release the destructive enzyme (for example, proteinases) in a heating-driven manner, should be very promising.

One more problem important for biological and medicinal use of a polymer-based complex as carrier vectors is its biocompatibility and biodegradability. Though it is not a trivial question how PNAGA can be degraded after enzyme delivery and release, polymers with a mass less than 40 kDa (which is true for short PNAGA-based copolymers with phase transition at physiological temperature) can be expected to be filtered by the kidneys [37]. Some clearance from PNAGA-based hydrogel was shown in [38]. In addition, PNAGA does not exhibit significant toxicity according to previous works [21,39]. However, the biocompatibility and biodegradability of such polymers require additional direct studies.

## 5. Conclusions and Perspectives

A thermoresponsive polymer, poly(*N*-acryloyl glycinamide), which is soluble in water at elevated temperatures but phase-separates at low temperatures, is shown to capture lysozyme at temperature lower than 10 °C and form stable polymer–enzyme complexes. Heating to room temperature (around 25 °C) resulted in the complex dissociation and release of the enzyme. Being almost inactive in a complexed form, lysozyme restored its enzymatic activity after a thermocontrolled release. In addition, capturing by the polymer partially protected lysozyme against proteolytic degradation, which is useful for biotechnological application. The reversible capturing-inactivation with a thermocontrolled release is promising for the medicinal use of poly(*N*-acryloyl glycinamide)-based polymers as drug vehicles to deliver enzyme-based therapeutics. The development of particular carriers with optimal phase-transition behavior should be an issue of future research, and our results taken together with the data on tuning phase transition temperature of the polymer by other groups suggest an outstanding potential for such carriers. In addition, such polymers can be used as a protein-capturing part of complex carrier combined with tags for targeted drug delivery. The biodegradability of the developed polymeric carriers also should be investigated directly.

## Data Availability

Data available on request.
